# Case report: a rare case of primary lung tumour with local intra-atrial invasion

**DOI:** 10.1093/ehjcr/ytaf591

**Published:** 2025-11-25

**Authors:** Mahmoud Eldesouky, Jeremy Butts, Ahmed Kattout, Karthik Viswanathan

**Affiliations:** Cardiology Department, Calderdale and Huddersfield NHS Foundation Trust, Halifax HX3 0PW, UK; Cardiology Department, Calderdale and Huddersfield NHS Foundation Trust, Halifax HX3 0PW, UK; Cardiology Department, Calderdale and Huddersfield NHS Foundation Trust, Halifax HX3 0PW, UK; Cardiology Department, Calderdale and Huddersfield NHS Foundation Trust, Halifax HX3 0PW, UK

**Keywords:** Cardiac MRI, Echocardiogram, Tumour, CT scan, Case report

## Abstract

**Background:**

Cardiac tumours are rare, with the majority arising as metastases from non-cardiac sources. Lung cancer is among the more frequent malignancies to metastasize the heart, but direct invasion of the left atrium via the pulmonary veins remains uncommon. Differentiating between benign tumours, thrombus, and malignant extension can be challenging, and multi-modality imaging plays a key role.

**Case summary:**

A 69-year-old woman with chronic obstructive pulmonary disease and hypertension was under investigation for a suspected primary lung malignancy with adrenal metastases. Computed tomography (CT) of the thorax demonstrated a possible left atrial thrombus. Subsequent transthoracic echocardiography revealed a large, mobile left atrial mass, initially thought to represent an atrial myxoma due to its stalk-like appearance and prolapse into the mitral valve. However, review of cross-sectional imaging raised concern for malignant invasion. Cardiac magnetic resonance imaging (MRI) confirmed a right middle lobe lung mass with direct extension into the right upper pulmonary vein and left atrium. The absence of atrial wall attachment was atypical for myxoma and supported tumour invasion. The patient was managed with best supportive care following multi-disciplinary discussion and sadly passed away within 6 months.

**Discussion:**

This case highlights the diagnostic challenges of intracardiac masses and emphasizes the importance of integrating echocardiography with advanced imaging modalities.

Learning points

- Demonstrating the added value of multi-modality imaging while evaluating intracardiac masses
- Highlighting the importance of reviewing and understanding the wider clinical context when interpreting individual cardiac imaging reports

## Introduction

A left atrial mass on transthoracic echocardiography was initially assessed to be an atrial myxoma. Further imaging with cardiac magnetic resonance imaging (MRI), correlated with clinical findings, revealed that the mass represented direct continuation of a primary pulmonary tumour into the left atrium via the right upper pulmonary vein.

## Summary figure

**Figure:**
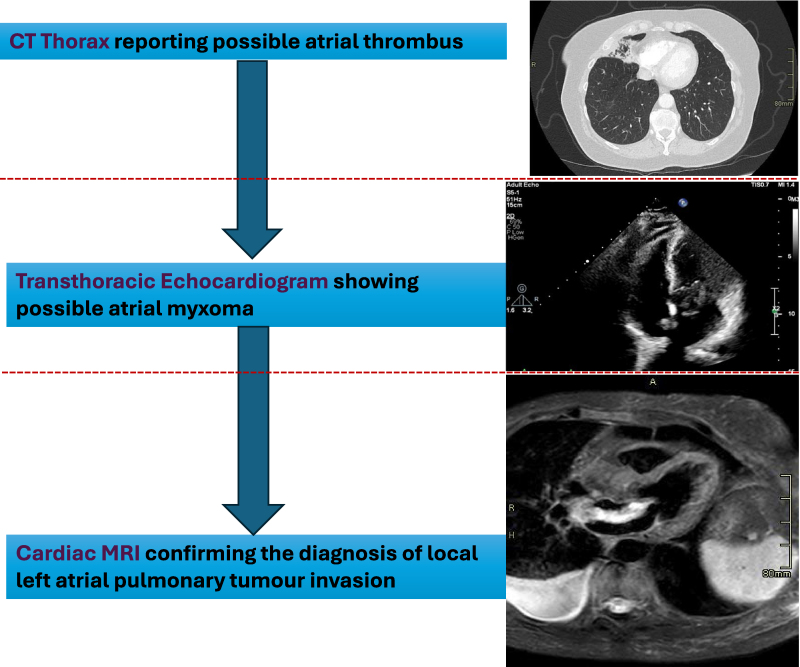


## Case description

**Figure 1:**
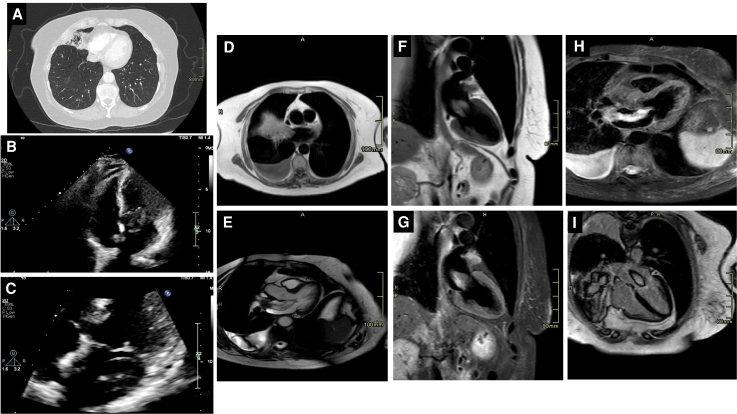
(*A*) The initially performed CT thorax showing what initially appeared to be an intra-atrial thrombus. (*B*) Transthoracic echocardiogram four-chamber left apical view showing a large left atrial highly mobile mass (30 × 17 mm) appearing to be attached by a stalk to the interatrial septum partially protruding into the left ventricle through the mitral valve. (*C*) Focused view of the left atrial mass described in *B*. (*D*) Cardiac MRI imaging (horizontal long axis) demonstrating a large right middle lobe lung mass with direct continuity into the right upper pulmonary vein and extension into the left atrium. The absence of discrete atrial wall attachment argues against myxoma. (*E*) Cardiac MRI horizontal plane with early and late gadolinium enhancement, showing no contrast uptake within the left atrial component of the mass. Lack of enhancement excludes organized thrombus (which typically demonstrates peripheral enhancement) and supports malignant extension. (*F*) Cardiac MRI vertical long axis view showing the intracardiac mass prolapsing into the mitral valve in diastole. The smooth prolapse without obstruction and absence of atrial wall tethering further distinguish tumour extension from myxoma. (*G*) Cardiac MRI T1- and T2-weighted sequences of the left atrial mass demonstrating intermediate T1 and high T2 signal intensity, with no suppression on fat-saturated imaging—features consistent with soft-tissue tumour rather than thrombus. (*H*) Cardiac MRI horizontal plane imaging showing mass continuity from the lung into the left atrium via the pulmonary vein. Despite diastolic prolapse across the mitral valve, there was no functional valve compromise. (*I*) Cardiac MRI late gadolinium enhancement sequence demonstrating absence of uptake within the mass, helping to exclude thrombus (expected peripheral enhancement) and supporting tumour invasion as the most likely diagnosis.

A 69-year-old woman with a history of chronic obstructive pulmonary disease and hypertension was under review by the lung multi-disciplinary team (MDT). She was an active smoker, and her only regular medication was amlodipine. There were no significant occupational or environmental risk factors, and she had no relevant family history. She initially presented with cough and right-sided chest pain, treated as community-acquired pneumonia. Ongoing consolidation on chest X-ray prompted referral to the MDT. Computed tomography (CT) imaging demonstrated a primary lung malignancy with adrenal metastases and a ‘possible atrial thrombus’ (*Figure 1A*). Physical examination, electrocardiography (ECG), and routine bloods were unremarkable.

An urgent outpatient echocardiogram arranged by the MDT revealed a large, highly mobile left atrial mass (30 × 17 mm) appearing to be ‘attached by a stalk to the interatrial septum’ partially protruding into the left ventricle through the mitral valve (*Figure 1B* and *C*; *Video 1*). Both atria were normal in size. Biventricular size and systolic function were preserved, with an estimated left ventricular ejection fraction of more than 55%. The mitral valve appeared structurally normal, with no evidence of flow obstruction and only trivial regurgitation. The atrial mass was initially described by the reporting cardiac physiologist as a ‘possible myxoma given the classical appearance’ on the echocardiogram images.

The cardiology team was consulted the same day. On review of the echocardiogram and CT, intracardiac invasion of the primary lung tumour was suspected, and urgent cardiac MRI was performed.

Cardiac MRI was conducted using a standard mass/tumour protocol, including cine images in multiple planes, early and late gadolinium enhancement, T1- and T2-weighted images with and without fat suppression, and first-pass perfusion. This revealed a large irregular right middle lobe lung mass (66 × 56 × 65 mm) with direct extension into the right upper pulmonary vein and subsequently into the left atrium. The intracardiac component measured ∼61 × 7 mm (*Figure 1D–I*; *Videos 2 and 3*). Findings were atypical for myxoma, due to proximal lung infiltration and lack of atrial wall attachment. There was no convincing fat suppression, first-pass perfusion, or contrast uptake on early or late gadolinium enhancement.

This conclusion was critical for both the cardiothoracic surgery and lung MDTs. In line with the patient’s wishes, she was commenced on best supportive care and sadly passed away within 6 months. No *trans*-oesophageal echocardiogram, biopsy, or autopsy was performed.

## Discussion

Cardiac tumours are rare, most representing metastases from non-cardiac sources.^1,2^ Approximately 8%–10% of lung cancers metastasize to the heart.^3^ This can happen via direct expansion and haematogenous or lymphatic spread.^2,4^ Direct left atrial invasion through pulmonary veins is rare, but has been reported in the past.^4–9^ In some cases, patients present with neurological symptoms secondary to embolic stroke.^2,4,8^ Others remain asymptomatic, and underdiagnosis is suspected, as cardiac imaging is not routinely performed.^5,10^ Better identification of these patients is valuable given that cardiac invasion is an independent poor prognostic marker in lung cancer.^11^

Our case demonstrates how a stepwise, multi-modality imaging strategy substantially altered the working differential and management. Initial suspicion of atrial thrombus following the CT scan led to transthoracic echocardiography, which strongly mimicked atrial myxoma and prompted surgical consideration. Cardiac MRI clarified the diagnosis by demonstrating direct tumour invasion. This conclusion was critical for both the cardiothoracic surgery and lung MDTs.

## Conclusion

This case highlights the value of multi-modality imaging in intracardiac mass evaluation. It underscores the need to interpret cardiac imaging in clinical context, particularly in patients with known malignancy. Recognition of tumour invasion is critical as it significantly influences prognosis and management.

## Lead author biography


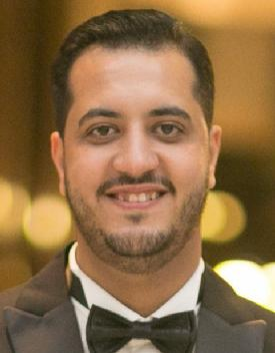


Mahmoud Eldesouky is a Cardiology Research Fellow at the University of Leicester and an Honorary Cardiology Fellow at Glenfield NHS Hospital. He started his career as a cardiothoracic intensive care unit (ICU) resident at Ain Shams University Hospitals, Egypt. He joined the NHS Workforce in 2018 and completed his Internal Medicine Training in 2022. He worked as a Cardiology Registrar in Mid Yorkshire NHS Hospitals and Calderdale & Huddersfield NHS Hospitals between 2022 and 2024.

## Data Availability

The data underlying this article will be shared on reasonable request to the corresponding author.

## References

[1] Alahmadi MH, Rodriguez Ziccardi M, Tariq MA, Limaiem F, Ahmed SW. Cardiac cancer. StatPearls [Internet] 2025. 30725829

[2] Chandra R, Abugroun A, Goldberg A, Cooney E, Mehrotra S, Volgman A. Small cell lung cancer invading the left atrium with subsequent malignant embolic stroke: a case report and review of literature. Cardiol Res 2019;10:188–192. 31236182 10.14740/cr752w PMC6575106

[3] Raza R, Mahabir R, Levi G. 7 squamous cell carcinoma of the lung-invading myocardium: an autopsy finding. Am J Clin Pathol 2018;149:S3–S3.

[4] Falanga G, Musumeci O, Stilo C, Zito C. Extension to the heart of metastatic lung cancer presenting as acute neurological syndrome: the key role of echocardiography. J Cardiovasc Echography 2014;24:89. 10.4103/2211-4122.143982 PMC5353445 28465913

[5] Dupas JY, Roudaut R, Vergeret J, Chevalier JM, Taytard A, Dallocchio M. [Left atrial extension of bronchial cancer diagnosed by echocardiography. Report of 2 cases]. Arch Mal Coeur Vaiss 1990;83:1593–1596. 2122836

[6] Lestuzzi C, Viel E, Mimo R, Meneguzzo N. Left atrial invasion by lung carcinoma through a pulmonary vein. Int J Cardiovasc Imaging 2001;17:107–110. 11558969 10.1023/a:1010621320976

[7] Chan V, Neumann D. Small cell lung carcinoma invading the pulmonary vein and left atrium as imaged by PET/CT. Eur J Nucl Med Mol Imaging 2005;32:1493–1493. 16180031 10.1007/s00259-005-1930-9

[8] Dimitrović A, Breitenfeld T, Supanc V, Roje-Bedeković M, Butković Soldo S, Vargek-Solter V. Stroke caused by lung cancer invading the left atrium. J Stroke Cerebrovasc Dis 2016;25:e66–e68. 26922131 10.1016/j.jstrokecerebrovasdis.2015.12.043

[9] Zhang T, Han Z, Hou S, Song Y, Zhang Y, Wang M. Metastatic small-cell lung carcinoma infiltrating the heart: a rare case diagnosed using imaging data. J Clin Ultrasound 2025;53:385–387. 39364805 10.1002/jcu.23853

[10] Roman S, Fichadiya H, Rushdy A, AbuArqob S, Bhavsar M, Noori MAM, et al The great invasion, a case of lung mass invading the heart through the pulmonary veins. Radiol Case Rep 2022;17:3219–3223. 35814818 10.1016/j.radcr.2022.06.025 PMC9256550

[11] Zhong Y, Li C, Sheng Y, Wang J, Wang G. Prognostic implication of direct cardiac invasion from lung cancer in non-operatively treated patients based on lung computed tomography imaging. Heart Lung Circ 2022;31:733–741. 34840061 10.1016/j.hlc.2021.10.018

